# Ketoconazole as second-line treatment for Cushing’s disease after transsphenoidal surgery: systematic review and meta-analysis

**DOI:** 10.3389/fendo.2023.1145775

**Published:** 2023-05-08

**Authors:** Camila Viecceli, Ana Carolina Viana Mattos, Vânia Naomi Hirakata, Sheila Piccoli Garcia, Ticiana da Costa Rodrigues, Mauro Antônio Czepielewski

**Affiliations:** ^1^ Graduate Program in Medical Sciences: Endocrinology, Faculty of Medicine, UFRGS, Porto Alegre, Brazil; ^2^ Endocrinology Division, Hospital de Clínicas de Porto Alegre, Porto Alegre, Brazil; ^3^ Federal University of Rio Grande do Sul (UFRGS), Porto Alegre, Brazil

**Keywords:** ketoconazole, Cushing’s disease, treatment, systematic review, meta-analysis

## Abstract

**Introduction:**

The first-line treatment for Cushing’s disease is transsphenoidal surgery for pituitary tumor resection. Ketoconazole has been used as a second-line drug despite limited data on its safety and efficacy for this purpose. The objective of this meta-analysis was to analyze hypercortisolism control in patients who used ketoconazole as a second-line treatment after transsphenoidal surgery, in addition to other clinical and laboratory criteria that could be related to therapeutic response.

**Methods:**

We searched for articles that evaluated ketoconazole use in Cushing’s disease after transsphenoidal surgery. The search strategies were applied to MEDLINE, EMBASE, and SciELO. Independent reviewers assessed study eligibility and quality and extracted data on hypercortisolism control and related variables such as therapeutic dose, time, and urinary cortisol levels.

**Results:**

After applying the exclusion criteria, 10 articles (one prospective and nine retrospective studies, totaling 270 patients) were included for complete data analysis. We found no publication bias regarding reported biochemical control or no biochemical control (p = 0.06 and p = 0.42 respectively). Of 270 patients, biochemical control of hypercortisolism occurred in 151 (63%, 95% CI 50-74%) and no biochemical control occurred in 61 (20%, 95% CI 10-35%). According to the meta-regression, neither the final dose, treatment duration, nor initial serum cortisol levels were associated with biochemical control of hypercortisolism.

**Conclusion:**

Ketoconazole can be considered a safe and efficacious option for Cushing’s disease treatment after pituitary surgery.

**Systematic review registration:**

https://www.crd.york.ac.uk/prospero/#searchadvanced, (CRD42022308041).

## 1 Introduction

Cushing’s disease (CD) results from an adrenocorticotropic hormone (ACTH) secreting pituitary tumor, which leads to chronic hypercortisolism (1, 2). It is a potentially fatal disease, with mortality rates up to 3.7 times higher than the general population (3, 4). CD is three times more common in women.

According to consensus, the first-line treatment for CD is pituitary tumor resection surgery with the transsphenoidal technique (4, 5), which achieves short-term biochemical control rates of 60 to 80%, depending on the experience of the treatment center. In long-term follow-up, recurrence rates range from 20 to 30% even in cases with complete initial biochemical control (6, 7).

Medication is a therapeutic option in patients who do not achieve biochemical control with transsphenoidal surgery (TSS), have recurrent hypercortisolism, and have contraindications or high surgical risk, or it can be used while waiting for the efficacy of radiation techniques (8). In such cases, adrenal-blocking drugs become important.

Ketoconazole is an antifungal drug, a synthetic imidazole derivative that blocks multiple enzymes involved in adrenal steroidogenesis pathways (CYP11A1, CYPP17, CYP11B2, and CYP11B1). It was recently approved for use in CD by the European Union (9) and has been recommended for off-label use in the United States (2, 10, 11). Although recommended by professional guidelines (not regulatory authorities) for hypercortisolism, its use as an antifungal has been more restricted since regulatory agencies in Europe and the United States have issued statements regarding its high risk of hepatotoxicity, including reported deaths from liver failure (12, 13). Recently, a levorotatory derivative (Levoketoconazole) with estimated lower hepatotoxicity was introduced (14).

Clinical studies evaluating the efficacy and adverse effects of ketoconazole in CD are scarce. Their limited and heterogeneous samples include hypercortisolism control as a first-line therapy or after TSS and they include patients with ACTH-dependent Cushing’s syndrome with indeterminate etiology (11–13).

Two recent meta-analyses had divergent results regarding hypercortisolism remission rates with ketoconazole use: 46% *vs*. 64% (15, 16). Adverse effects, treatment interruption, and treatment-associated deaths have also been reported. Thus, studies evaluating the efficacy of ketoconazole for its main indication and continued or recurrent hypercortisolism after TSS are not currently available.

This meta-analysis aimed to analyze the prevalence of biochemical control of hypercortisolism in CD patients who used ketoconazole as a second-line therapy after TSS, in addition to clinical and laboratory parameters that can predict therapeutic response and serious adverse effects due to ketoconazole treatment.

## 2 Materials and methods

This systematic review and meta-analysis study was performed according to the PRISMA system (17) and was registered in the International Prospective Register of Systematic Reviews (CRD42022308041).

### 2.1 Identification of studies

A search was performed in three databases: MEDLINE, EMBASE, and SciELO. In MEDLINE, using the Medical Subject Headings “Pituitary ACTH hypersecretion” or “Cushing’s disease” and “Ketoconazole” or “Fluconazole”, 305 articles were found. In EMBASE, using the Emtree terms “Cushing’s disease” and “ketoconazole” or “fluconazole”, 544 results were found. In SciELO, using the terms “Cushing’s disease” and “Ketoconazole” or “fluconazole”, five articles were found.

The complete search strategy can be found in **Supplementary Material 1**. The searches were performed in June 2021 and updated in May 2022 although no new studies were added to the analysis through this step. A manual search was performed for references to reviews and meta-analyses in the included studies, as well as systematic reviews or articles on related topics. Every potential article was considered eligible for review, with no language limitations. Whenever necessary, authors were contacted to confirm information or supply missing data.

### 2.2 Selection criteria

We selected observational, case-control, or clinical trials that included CD patients diagnosed through clinical manifestations in association with at least two positive screenings for hypercortisolism, baseline ACTH > 20 pg/ml, pituitary adenoma confirmed in surgery, bilateral petrosal sinus catheterization, or pituitary MRI showing a lesion > 6 mm (18). Patients must have undergone transsphenoidal surgery as first-line therapy, either without postoperative remission or with recurrence during clinical follow-up. Consequently, ketoconazole was used as a second-line treatment to control hypercortisolism. Studies of patients who received radiotherapy concomitantly with ketoconazole were not excluded.

### 2.3 Study selection, data extraction, and quality assessment

Two authors (CV and ACVM) performed independent searches in the databases, selecting potential studies based on titles and abstracts for further analysis of the complete articles. Inter-rater agreement was 0.88 according to Cohen’s kappa coefficient (95% CI, 0.83-0.93) for the selected studies. Disagreements were resolved by consensus between the investigators (CV and ACVM) or when necessary, by a discussion with a third investigator (MAC). Baseline characteristics and outcomes were extracted from studies that met the inclusion criteria, including baseline and post-drug cortisol measurements, mean and maximum treatment duration, ketoconazole dose, potential adverse effects, and drug intolerance. The considered outcomes were the prevalence of complete, partial (reduction of > 50% in cortisol levels despite incomplete normalization of 24-h UFC), or no biochemical control of hypercortisolism with ketoconazole use.

Data were extracted only when the studies reported ketoconazole use after transsphenoidal surgery (TSS). Studies that did not subdivide ketoconazole data into pre-and post-transsphenoidal surgery were excluded.

Disagreements about data extraction were discussed until a consensus was reached. The original authors were contacted by e-mail to resolve questions or obtain missing data. Study quality was evaluated using a modified Newcastle–Ottawa scale (19).

### 2.4 Data analysis

Rates of complete, partial, and no biochemical control were analyzed across all included studies and the pooled prevalence was calculated. Cochrane’s χ^2^ and I² tests were used to assess heterogeneity between studies, and p = 0.05 was considered significant. Incidence estimates were obtained by random effects models. Meta-regression was performed to analyze the relationship between ketoconazole dose, treatment time, and baseline cortisol level.

Publication bias was assessed with a funnel plot that assesses the incidences in relation to the standard error of each study, which was determined using the Begg and Egger tests. Meta-analysis was performed using R version 4.1.2 and R META package version 4.19.2.

## 3 Results

**Figure 1:**
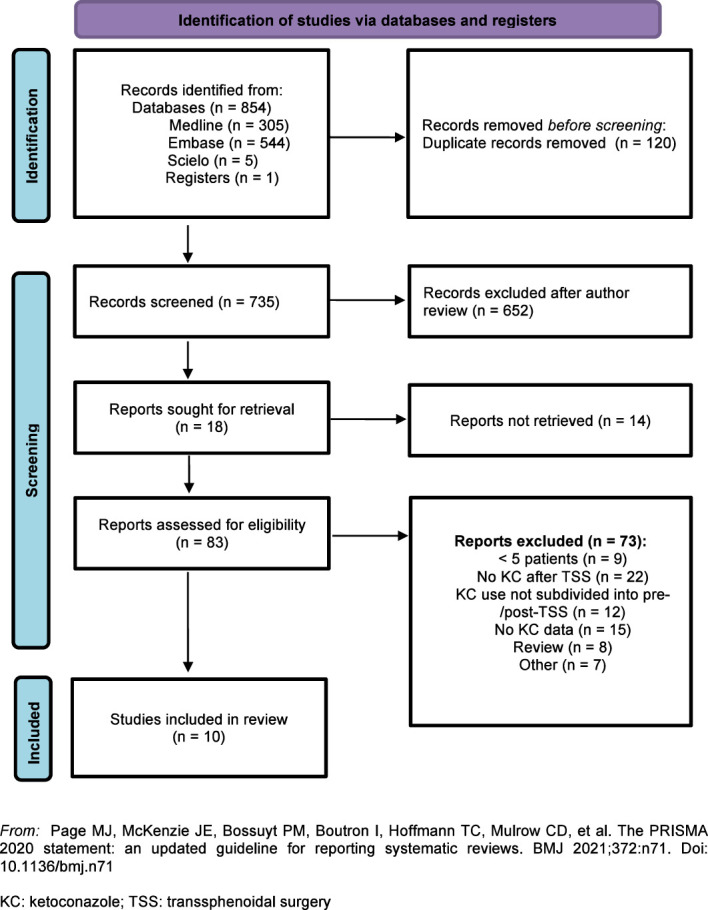
Flow diagram: Identification and selection of articles for the meta-analysis.

Electronic and manual database searches resulted in 735 studies, of which 652 were excluded after analyzing the titles and abstracts. We selected 83 studies for full-text review. After applying the exclusion criteria, 10 articles remained (totaling 270 patients) for analysis and complete data extraction (10, 20–28). The flow diagram is shown in **Figure 1**. No articles using the term fluconazole in the context of CD were found in the searches.

All of the selected studies used normalized 24-h UFC levels as a criterion for biochemical control of hypercortisolism except for one (24), which used serum cortisol level and the suppression test with 2 mg of dexamethasone (Liddle test).

Most patients were women and were treated with ketoconazole for a mean of 31.4 months and a maximum of 45 months. Details of each included study are presented in **Table 1**. Unpublished data from a conference abstract from a Brazilian cohort were included and were supplemented through direct contact with the authors (27).

**Table 1:** Characteristics of the included studies.

Author/Year	Title	Study design	Country	Study period	Total N with CD		N who used KC post-TSS	Remission criterion	Sex (F/M)	Mean treatment duration, months	Maximum treatment duration, months
Castinetti F 2008	Ketoconazole revisited: a preoperative or postoperative treatment in Cushing’s disease.	Single-center retrospective cohort	France	1995-2005	38	17		24-h UFC	14/3	22.9	72
Castinetti F 2014	Ketoconazole in Cushing’s disease: is it worth a try?	Multicenter Retrospective Cohort (14 centers)	France	1995-2012	200	141		24-h UFC	NA	24.8	135
Di Somma C 1998	Effectiveness of chronic treatment with alendronate in the osteoporosis of Cushing’s disease.	Prospective cohort	Italy	NA	39	18		24-h UFC	8/10	NA	12
Espinosa de los Monteros 2017	Long-term outcome of the different treatment alternatives for recurrent and persistent Cushing disease.	Single-center retrospective cohort	Mexico	1991 - 2014	89	13		24-h UFC	13/0	26	47
Huguet I 2015	Assessment of the outcomes of the treatment of Cushing’s disease in the hospitals of Castilla-La Mancha.	Multicenter Retrospective Cohort (6 centers)	Spain	2000 - 2012	22	5		24-h UFC	NA	NA	NA
Kakade HR 2014	Clinical, biochemical, and imaging characteristics of Cushing’s macroadenomas and their long-term treatment outcome	Single-center retrospective cohort	India	1997 - 2013	232	6		Serum cortisol/Liddle test	NA	NA	NA
Luisetto G 2001	Recovery of bone mineral density after surgical cure, but not by ketoconazole treatment, in Cushing’s syndrome.	Single-center retrospective cohort	Italy	NA	13	10		24-h UFC	9/1	44.9	100
Sonino N 1991	Ketoconazole treatment in Cushing’s syndrome: experience in 34 patients	Single-center retrospective cohort	Italy	NA	28	18		24-h UFC	15/3	3.8	12
Viecceli C 2022	Evaluation of ketoconazole as a treatment for Cushing’s disease in a retrospective cohort	Single-center retrospective cohort	Brazil	2004 - 2020	172	26		24-h UFC	22/4	65.7	173
Correa Silva SR 2011	Preoperative and long-term postoperative ketoconazole treatment in Cushing’s Disease: Clinical aspects and plasma ACTH behavior during its use	Single-center retrospective cohort	Brazil	NA	32	16		24-h UFC	13/3	31.5	81

CD, Cushing’s disease; KC, ketoconazole; NA, not available; TSS, transsphenoidal surgery; 24-h UFC,: 24-h urinary free cortisol.

The study quality analysis is shown in **Table 2**. In general, the quality of the articles was adequate. Some data could not be extracted due to uncertainty about when TSS had been performed and ketoconazole therapy had begun. In such cases, the authors were contacted and, if they did not respond by the time of the analyses, the data were excluded. The study by Huguet et al. (23) was excluded from the analysis of the “no biochemical control” variable for not mentioning non-remission as a possible outcome.

**Table 2:** Quality of the included studies (one-star maximum for each item, except comparability of cohorts, with two maximum).

Author	Representativeness of the exposed cohort	Selection of non-exposed cohort	Ascertainment of exposure	Outcome not present at the start of the study	Comparability of cohorts	Assessment of outcomes	Follow-up length	Adequacy of follow-up
Castinetti F, 2008	★			★	★	★	★	★
Castinetti F, 2014	★			★	★	★	★	★
Di Somma C. 1998	★		★	★	★	★	★	★
Espinosa de los Monteros, 2017	★	★	★	★	★	★	★	★
Huguet I, 2015	★	★		★	★	★	★	
Kakade HR, 2014	★	★		★	★	★	★	
Luisetto G, 2001	★			★	★★	★	★	★
Sonino N, 1991	★			★	★	★	★	★
Viecceli C, 2022	★			★	★	★	★	★
Correa Silva SR, 2011^§^	★			★	★	★	★	★

^§^ Only abstracts available for evaluation. The star is the symbol used in this type of quality table.

**Figure 2:**
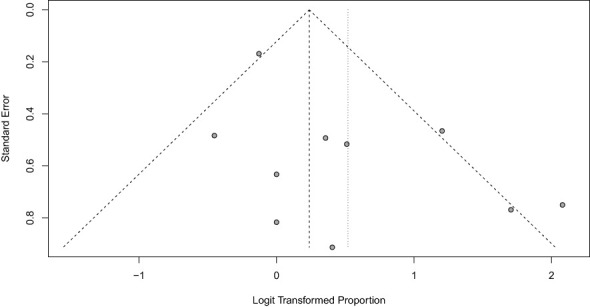
Funnel Plot of hypercortisolism remission with Ketoconazole.

**Figure 3:**
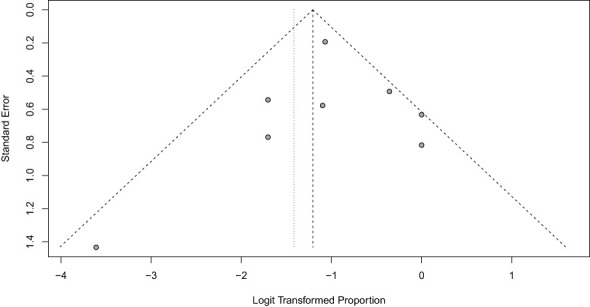
Funnel Plot of hypercortisolism non-remission with ketoconazole.

Begg and Egger’s tests were performed to assess publication bias regarding biochemical control of hypercortisolism. Since the results were not significant, there was no need to perform a trim-and-fill analysis. Funnel Plots (**Figures 2**, **3**) demonstrate the lack of publication bias regarding biochemical control and no biochemical control (p = 0.06 and p = 0.42, respectively).

### 3.1 Control of hypercortisolism (biochemical control)

**Figure 4:**
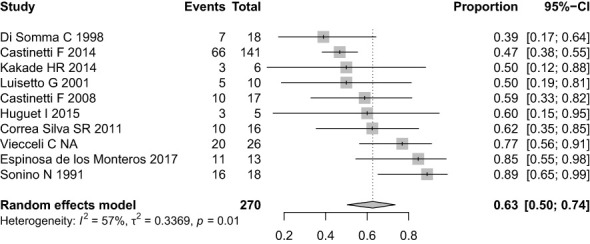
Forest plot of hypercortisolism remission with Ketoconazole.

Ten studies (270 patients) indicated the prevalence of biochemical control of hypercortisolism in patients who underwent TSS and received ketoconazole as a second-line therapy. A total of 151 patients had complete biochemical control (63%; 95% CI, 50-74%; see **Figure 4**). We performed a meta-analysis without including Correa Silva’s unpublished data, and the prevalence of hypercortisolism remission remained at 63%. These charts can be found in the **Supplementary Material**.

The high variability between studies is partly explained by the clinical differences between cohorts, which explain the 39 to 89% variation in remission rates. The lowest complete remission rate, 39%, was found in Di Somma et al. However, in addition to being the only prospective study, there was a high rate of partial biochemical control (61%), and no patient was classified as no biochemical control. This cohort also had the highest mean baseline cortisol levels (1413 nmol/24h, 9.46 times above the upper reference limit) and the lowest mean final ketoconazole dose (400 mg daily). The highest remission rate, 89%, was found in Sonino et al., a retrospective cohort, which might explain why ketoconazole was administered only in patients with a more favorable clinical response. Heterogeneity was 57% in this analysis.

**Figure 5:**
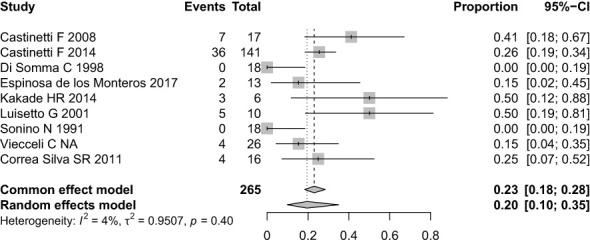
Forest plot of hypercortisolism non-remission with ketoconazole.

No biochemical control occurred in 61 of 270 patients or 20% of the sample (95% CI, 10-35%) (**Figure 5**). The four cohorts with the highest rates of non-remission, Kakade HR et al. (50%), Luisetto G et al. (50%), Castinetti F et al. (41%), and Espinosa de los Monteros et al. (26.7%) did not involve the concept of partial biochemical control, which was used in the other cohorts. Heterogeneity was 4% in this analysis.

Although the concept of partial response was not addressed directly in most studies, some patients experienced a reduction of > 50% in cortisol levels despite incomplete normalization. This condition was described in five cohorts (10, 21, 26, 27, 28), demonstrating partial benefits from ketoconazole in 59 patients (21.7%).

Only five papers mentioned how many patients underwent radiotherapy during treatment with ketoconazole; at least 59 patients (21%) received radiotherapy treatment concomitantly or subsequent to ketoconazole (10, 22, 23, 27, 28).

### 3.2 Adverse effects

Although all of the studies described adverse effects from ketoconazole, only two provided information about them after TSS (26, 28). The following stood out among the main adverse effects: elevated transaminase levels, diarrhea, abdominal pain, skin rash, gynecomastia, and adrenal insufficiency. Medication discontinuation due to intolerance was reported in three studies (10, 20, 28). Due to insufficient data, it was not possible to perform a meta-analysis of the prevalence of adverse effects. No deaths related to ketoconazole were reported in any study.

### 3.3 Meta-regression

In studies that evaluated hypercortisolism remission, meta-regression was used to analyze which variables influenced the occurrence or not of biochemical control. Both the final dose of ketoconazole (six studies with a mean dose of 628 mg/day: range 400 mg to 779 mg/day), the duration of drug treatment (seven studies with a mean duration of 31 months), and the baseline 24-h UFC levels (seven studies with a mean of 4.48 times above the reference value) showed no association with hypercortisolism remission (data not shown).

## 4 Discussion

Drug treatment in CD is reserved only for patients with no biochemical control after TSS, in those who are not candidates for surgical treatment, or in those awaiting the effects of radiotherapy (2, 4). The available drugs in this context act in several ways: as adrenal blockers (ketoconazole, osilodrostat, metyrapone, mitotane, levoketoconazole, and etomidate), somatostatin receptor ligands (pasireotide), dopamine receptor agonists (cabergoline), or as glucocorticoid receptor blockers (mifepristone) (2, 29). These drugs must be prescribed considering aspects such as the potential for remission, potential adverse effects, availability, and cost. Moreover, no single drug has yet been demonstrated as superior to the others (2, 30, 31).

Comparing our analyses with previous studies, we found that hypercortisolism control in patients who had already undergone TSS was higher than in studies that did not subdivide ketoconazole use into pre- and post-transsphenoidal surgery or in studies evaluating multiple etiologies of hypercortisolism (15, 16, 32).

Our meta-analysis evaluated 10 studies from different countries and ethnic groups regarding CD treatment with ketoconazole due to non-remission or recurrence after TSS. The hypercortisolism biochemical control rate we found after TSS (63%) was greater than some prospective studies evaluating current drugs such as levoketoconazole but was also similar to that found in a systematic review by Pivonello et al. (64%) (14, 32). However, it was higher than that found in the most recent meta-analysis (36 to 46%) (15). These two systematic reviews (14, 15) did not subdivide ketoconazole use into pre- and post-transsphenoidal surgery, which can significantly impact the hypercortisolism control rate. A multicenter study by Castinetti et al. showed greater efficacy in patients who had already undergone TSS (68% control) compared to preoperative use (48.7% control) (10). These findings may be due to the fact that assessing patients with different states of hypercortisolism broadens the sample beyond only CD patients (i.e., probably including patients with ectopic ACTH syndrome and other etiologies) and, thus, the percentage of controlled patients may be lower.

According to the literature, even without complete biochemical control, patients who present some reduction in serum cortisol levels, partial biochemical control, or improvement in any associated comorbidities are candidates for continuing ketoconazole alone or in a possible association with other medications (2). Our meta-analysis found that such was the case in 59 patients. Although the concept of partial response was not addressed directly in most of the included studies, some individuals experienced a > 50% reduction in cortisol levels but not complete normalization. By analyzing the overall rate of non-responders (20%), we can extrapolate that approximately 80% of patients treated with ketoconazole experienced some improvement in cortisol levels, which in itself demonstrates the medication’s efficacy.

Although we considered the hypercortisolism biochemical control rate to be satisfactory with ketoconazole, many patients may lose biochemical control over the course of treatment or have long-term oscillations, and it has been suggested that this can occur in up to 23% of those who achieved initial control using the drug (2, 32), which shows the dynamic nature of their treatment and the constant challenge in clinical practice. This could not be established in our meta-analysis due to the lack of reported data (15, 16, 32). Although tumor size is not necessarily related to cortisol levels in CD, those with macroadenomas have a lower chance of remission after TSS (2, 33). Patients who use ketoconazole preoperatively may already have larger lesions, which makes surgery difficult, or active pituitary lesions, which can reduce the ability to achieve control through medication. In our meta-analysis, only two studies described tumor size and correlated it with remission after ketoconazole therapy (10, 24).

The hypothesis that patients with lower pre-treatment serum cortisol levels or who used higher doses of ketoconazole would have higher biochemical control rates was not confirmed since we found no relationship between longer duration of use and higher remission rates. The data included in this review do not provide a profile of patients most likely to benefit from ketoconazole treatment. Other reviews of ketoconazole therapy in any context of Cushing’s syndrome have found that up to 20% of patients experience adverse effects such as elevated transaminase levels, with the majority being asymptomatic moderate elevation, i.e., < 5 times the upper limit of normality. These hepatic changes do not appear dose-dependent and are usually reversed within 2 to 12 weeks after ketoconazole discontinuation or dose reduction (34). When compared, up to 32% of participants experienced mild adverse effects in the levoketoconazole study, with 13% having to discontinue treatment (14). Our analyses have several limitations since nine of the 10 primary studies that were included in the meta-analysis were retrospective and uncontrolled in design. We could find no randomized clinical trials, and we know that only randomized, controlled trials with an intention to treat analysis can provide accurate estimates of drug efficacy. New therapeutic options are under investigation in clinical trials and will likely bring more robust data about hypercortisolism control in CD.

Despite the limitations, consensus continues to indicate adrenal blockers, including ketoconazole, for patients with moderate CD and no visible lesions in MRI. The recommendation is that drug therapy should be individualized, based on the patient’s clinical picture, hypercortisolism severity, and medication availability and cost, so that treatment is optimized and applied for the necessary period of time (2, 33, 35, 36).

## 5 Conclusion

Our meta-analysis showed that ketoconazole effectively controlled hypercortisolism in approximately 63% of CD patients when used according to its principal indication, i.e., in patients without remission after TSS. No association was found between hypercortisolism biochemical control and total medication dose, treatment duration, or initial serum cortisol levels. No serious adverse effects or treatment-related deaths were observed in these patients. These findings indicate that based on the current literature available, ketoconazole is an efficacious and safe drug for treating active CD after pituitary surgery.

## Data availability statement

The original contributions presented in the study are included in the article/**Supplementary Material**. Further inquiries can be directed to the corresponding author.

## Author contributions

CV, SPG and MAC created the research format. CV and ACVM developed the search strategies and independently applied the eligibility criteria, subsequently extracting the data. CV and ACVM performed a peer review of the data and assessed risk of bias. CV and VNH performed the meta-analysis. MAC oversaw all phases of the meta-analysis and arbitrated conflicts of opinion. SPG and TCR participated in the final data review and discussion. All authors contributed to the article and approved the submitted version.
