# Supplementary figures and images for: Ketoconazole as second-line treatment for Cushing’s disease after transsphenoidal surgery: systematic review and meta-analysis
Source: Front Endocrinol (Lausanne). 2023 May 8;14:1145775. doi: 10.3389/fendo.2023.1145775 (PMC10200879; doi:10.3389/fendo.2023.1145775)

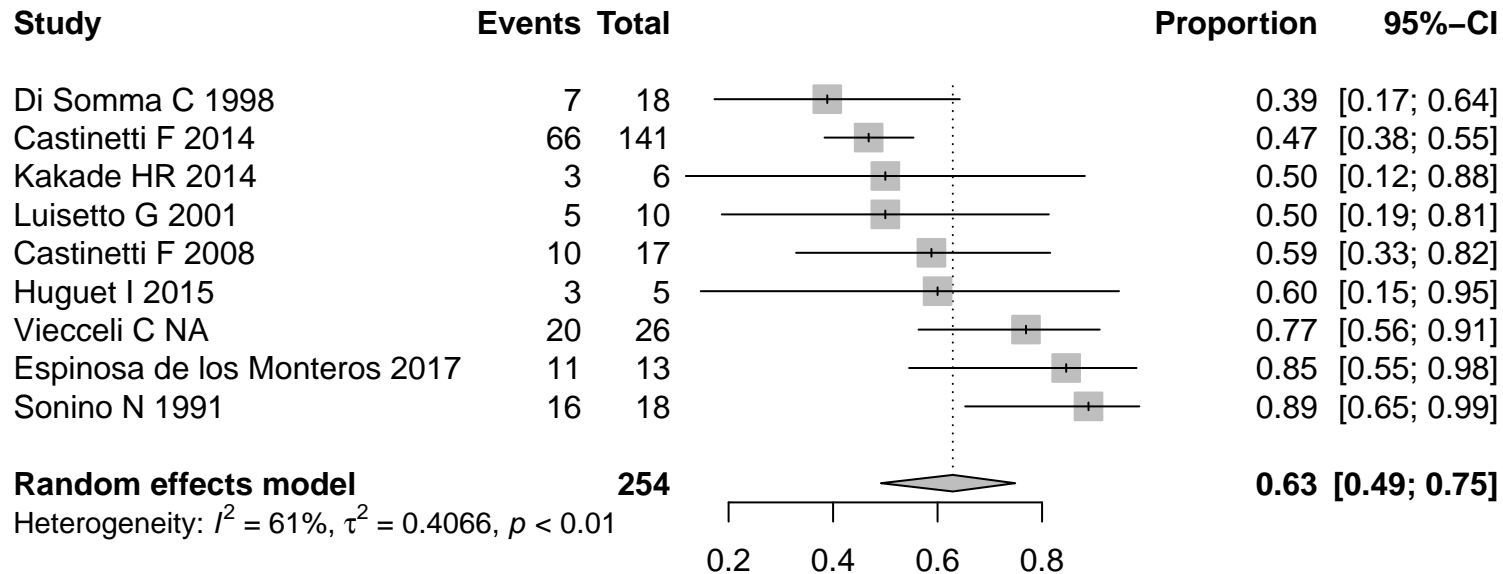

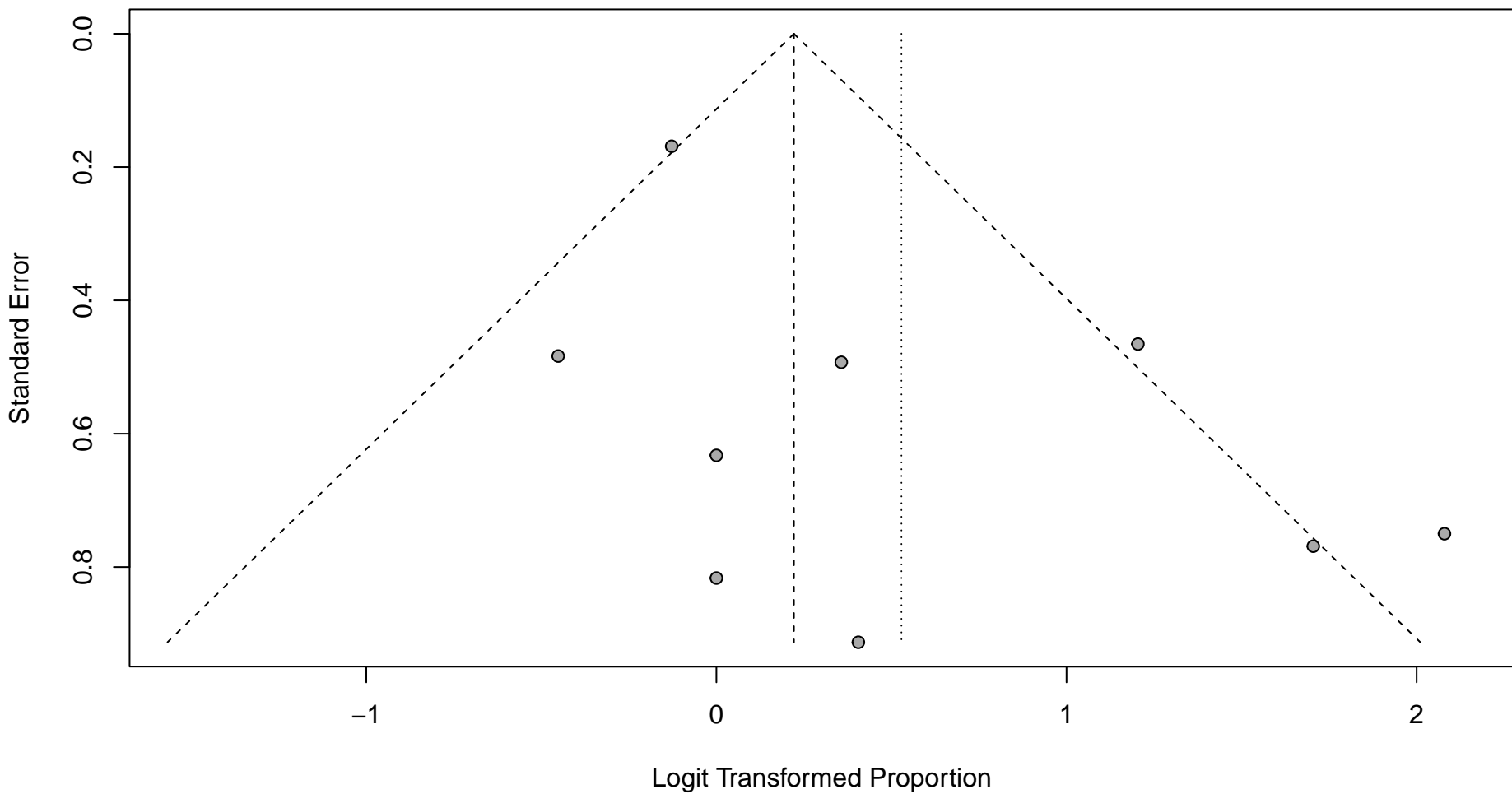

Supplement: Supplementary file 1 [file DataSheet_1.pdf]

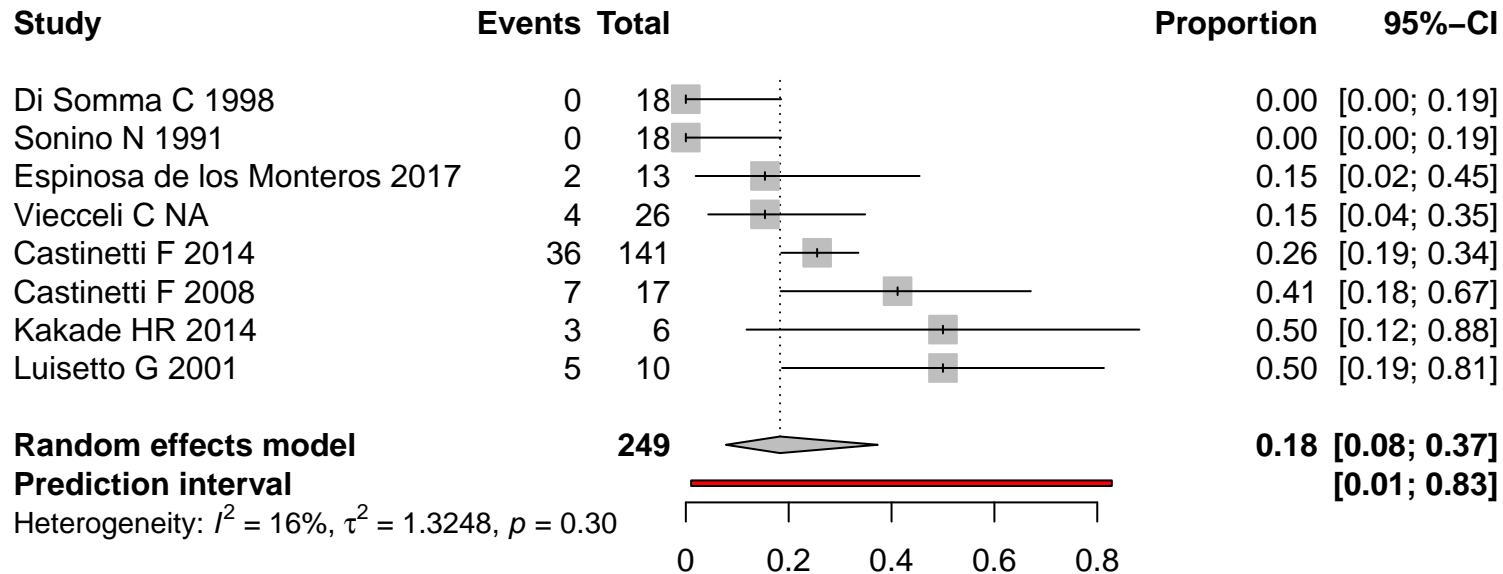

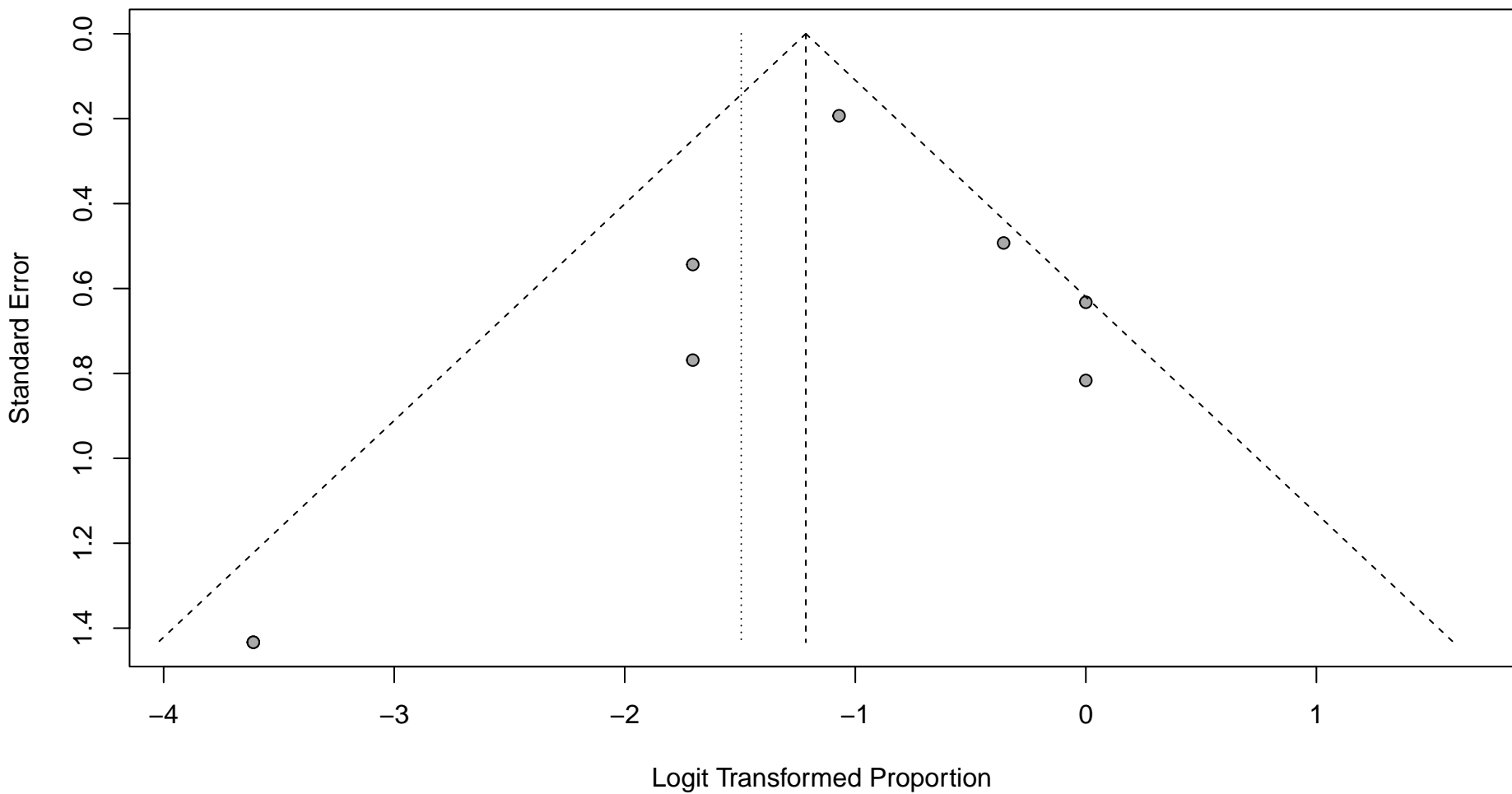

Supplement: Supplementary file 2 [file DataSheet_2.pdf]
