# Supplementary material for: Ketoconazole as second-line treatment for Cushing’s disease after transsphenoidal surgery: systematic review and meta-analysis
Source: Front Endocrinol (Lausanne). 2023 May 8;14:1145775. doi: 10.3389/fendo.2023.1145775 (PMC10200879; doi:10.3389/fendo.2023.1145775)
Supplement: Supplementary file 3 [file DataSheet_3.docx]

**Supplementary Material:**

Search strategies for all databases.

**MEDLINE search: 305 results**

(Pituitary ACTH Hypersecretion[mh] OR ACTH-Secreting Pituitary Adenoma[mh] OR Cushing Disease[tw] OR "Cushing's Disease"[tw] OR (Pituitary[tw] AND (ACTH Hypersecret*[tw] OR Hypercortisolism[tw] OR Cushing[tw] OR CS[tw] OR ACTH Secret*[tw] OR ACTH-Producing[tw] OR Adrenocorticotropic hormone-secret*[tw] OR adrenocorticotropic hormone-hypersecret*[tw] OR Adrenocorticotropic hormone-producing[tw] OR Corticotropin-Secreting Adenoma*[tw])) OR Inappropriate ACTH Secretion[tw] OR Inappropriate Adrenocorticotropic Hormone Secretion[tw] OR Adrenocorticotropic hormone-dependent Cushing[tw] OR ACTH-dependent Cushing[tw] OR Endogenous Cushing[tw] OR hypercortisolism[tw] OR Corticotroph Adenoma*[tw] OR Pituitary Neoplasm*[tw])

AND

(Ketoconazole[mh] OR Ketoconazole[tw] OR Nizoral[tw] OR R-41400[tw] OR "R41,400"[tw] OR fluconazole[mh] OR fluconazole[tw] OR

Diflucan[tw] OR Fungata[tw] OR Triflucan[tw] OR "UK 49858"[tw] OR "UK 49858"[tw] OR Zonal[tw])

**Embase search: 544 results**

('cushing disease'/exp OR 'acth secreting adenoma'/exp OR 'cushing disease':ti,ab,kw OR 'cushings disease':ti,ab,kw OR (pituitary:ti,ab,kw AND (acth AND hypersecret*:ti,ab,kw OR hypercortisolism:ti,ab,kw OR cushing:ti,ab,kw OR cs:ti,ab,kw OR 'acth secret*':ti,ab,kw OR 'acth-producing':ti,ab,kw OR 'adrenocorticotropic hormone-secret*':ti,ab,kw OR 'adrenocorticotropic hormone-hypersecret*':ti,ab,kw OR 'adrenocorticotropic hormone-producing':ti,ab,kw OR 'corticotropin-secreting adenoma*':ti,ab,kw)) OR 'inappropriate acth secretion':ti,ab,kw OR 'inappropriate adrenocorticotropic hormone secretion':ti,ab,kw OR 'adrenocorticotropic hormone-dependent cushing':ti,ab,kw OR 'acth-dependent cushing':ti,ab,kw OR 'endogenous cushing':ti,ab,kw OR hypercortisolism:ti,ab,kw OR 'corticotroph adenoma*':ti,ab,kw OR 'pituitary neoplasm*':ti,ab,kw) AND ('ketoconazole'/exp OR ketoconazole:ti,ab,kw OR nizoral:ti,ab,kw OR 'r 41400':ti,ab,kw OR 'r41,400':ti,ab,kw OR 'fluconazole'/exp OR fluconazole:ti,ab,kw OR diflucan:ti,ab,kw OR fungata:ti,ab,kw OR triflucan:ti,ab,kw OR 'uk-49858':ti,ab,kw OR 'uk 49858':ti,ab,kw OR zonal:ti,ab,kw)

**SciELO search: 5 results**

(subject:("Pituitary ACTH Hypersecretion" OR "ACTH-Secreting Pituitary Adenoma" OR "Cushing Disease" OR "Cushing's Disease" OR "cushing's syndrome")) AND (subject:(Ketoconazole OR Ketoconazole OR Nizoral OR R-41400 OR "R41,400" OR fluconazole OR fluconazole OR Diflucan OR Fungata OR Triflucan OR "UK-49858" OR "UK 49858" OR Zonal))
